# Supplementary material for: Application of In vitro transcytosis models to brain targeted biologics
Source: PLoS One. 2023 Aug 23;18(8):e0289970. doi: 10.1371/journal.pone.0289970 (PMC10446226; doi:10.1371/journal.pone.0289970)
Supplement: S1 Data — (DOCX) [file pone.0289970.s001.docx]

Supporting Data

Fig 1D

Transported mabs (ng)

Fig 1E

Transported mabs (ng)

Fig 2A

TEER value of individual transwells (ohm)

Fig 2C

Fig 2D hTfR EC50

Figure 2D cyno-TfR reactivity

Binding signal fold over mouse IgG control

Figure 2D Caco-2 transcytosis

Fig 3B

Fig 3C

Transported mabs (ng)

Fig 3D

Fig 3E

Fig 3F

Fig 4B

Transported mabs

Fig 5A

Fig 5C

Fig 6B

Fig 7
