# Supplementary material for: Application of In vitro transcytosis models to brain targeted biologics
Source: PLoS One. 2023 Aug 23;18(8):e0289970. doi: 10.1371/journal.pone.0289970 (PMC10446226; doi:10.1371/journal.pone.0289970)
Supplement: Attachment — Submitted filename: 2023 0623 response to Reviewers comments.docx [file pone.0289970.s002.docx]

Reviewers' comments:

Reviewer's Responses to Questions

**Comments to the Author**

1. Is the manuscript technically sound, and do the data support the conclusions?

The manuscript must describe a technically sound piece of scientific research with data that supports the conclusions. Experiments must have been conducted rigorously, with appropriate controls, replication, and sample sizes. The conclusions must be drawn appropriately based on the data presented.

Reviewer #1: Yes

Reviewer #2: Yes

2. Has the statistical analysis been performed appropriately and rigorously?

Reviewer #1: No

Reviewer #2: No

3. Have the authors made all data underlying the findings in their manuscript fully available?

The [PLOS Data policy](https://nam12.safelinks.protection.outlook.com/?url=http%3A%2F%2Fwww.plosone.org%2Fstatic%2Fpolicies.action%23sharing&data=05%7C01%7Ckatherine.deng%40abbvie.com%7Ca81a2fb6e58a4e6c672708db51cbaaa4%7C6f4d03de95514ba1a25bdce6f5ab7ace%7C0%7C0%7C638193707939002656%7CUnknown%7CTWFpbGZsb3d8eyJWIjoiMC4wLjAwMDAiLCJQIjoiV2luMzIiLCJBTiI6Ik1haWwiLCJXVCI6Mn0%3D%7C3000%7C%7C%7C&sdata=H1TUdgb5kADYOi46wluSE9CxhMuCIW5JPZut%2Fs7Ub5c%3D&reserved=0) requires authors to make all data underlying the findings described in their manuscript fully available without restriction, with rare exception (please refer to the Data Availability Statement in the manuscript PDF file). The data should be provided as part of the manuscript or its supporting information, or deposited to a public repository. For example, in addition to summary statistics, the data points behind means, medians and variance measures should be available. If there are restrictions on publicly sharing data—e.g. participant privacy or use of data from a third party—those must be specified.

Reviewer #1: Yes

Reviewer #2: Yes

4. Is the manuscript presented in an intelligible fashion and written in standard English?

PLOS ONE does not copyedit accepted manuscripts, so the language in submitted articles must be clear, correct, and unambiguous. Any typographical or grammatical errors should be corrected at revision, so please note any specific errors here.

Reviewer #1: Yes

Reviewer #2: Yes

5. Review Comments to the Author

Please use the space provided to explain your answers to the questions above. You may also include additional comments for the author, including concerns about dual publication, research ethics, or publication ethics. (Please upload your review as an attachment if it exceeds 20,000 characters)

Reviewer #1: The authors evaluated transcytosis of anti-TfR targeted antibodies in multiple in vitro transcytosis models. The authors suggest that high TEER value is a key parameter to differentiate RMT-mediated transcytosis from non-specific, paracellular transport of antibodies. The author also evaluated transcytosis of a panel of anti-hTfR antibodies in Caco-2 and MBT model. They also evaluated one of the antibodies in monkey in vivo PET study. Finally, they evaluated anti-TfR antibodies in a mouse model. Below are my comments/questions:
1. The authors need to do stats on all their findings to claim any significance or non significance.

The stats were added to all data.

2. In Figure 3, the number of replicates is only 2 for some of the panels. Please confirm as stats cannot be done on these findings.

PET studies were indeed performed in two NHPs for each radiolabeled antibody. This has now been clarified in the legend of Figure 3. We agree with the reviewer that it is not appropriate to perform statistical analysis on a dataset with this limited number of subjects. Protein-naïve NHPs are very challenging to access and studies are typically restricted to a limited sample size. However, data from PET measurements in NHPs has been shown highly reproducible and provide important translational value.

3. Throughout the text and figures, the authors mention the terms ‘brain concentration’ and ‘blood concentration’ several times especially for PET imaging. However, concentrations such as values in pg/mL or nM are not provided. If the authors have these values, please provide them. Otherwise, the correct the terms to ‘relative levels’.

PET is used to measure the amount of radioactivity per volume of blood (kBq/mL) or tissue (kBq/cc). We therefore prefer to use the term radioactivity concentration. The radioactivity concentration is expressed in the standardized uptake value (SUV) unit. SUV is a commonly used unit and includes normalization for the amount of injected radioactivity and the subject mass and thereby allows for direct comparison between subjects and across species. For further clarification, we have now updated relevant text to ‘radioactivity concentration’ throughout the manuscript.

4. In the results for PET imaging, the authors claim that the control IgG has higher blood concentrations than anti-hTfR.B1. Could this be due to different stability of the two antibodies in vivo? Has the PK of the two antibodies been compared in rodents?

There was no indication that the radiolabeled antibodies were unstable in vivo. The two antibodies were also conjugated and radiolabeled using the same conditions and methods. We did not compare the PK of these two antibodies in rodents. Anti-TfR antibodies are known to have fast clearance due to target-mediated drug disposition (TMDD) based on our internal data and literature. Anti-hTfR.B1 binds cyno TfR but not rodent TfR, thus is expected to exhibit fast clearance in cyno monkey but not in rodents due to TMDD. The control antibody has a longer half-life in blood (which is consistent with other experiments not included in this manuscript).

5. Why was the brain concentration corrected against the heart concentration (as is mentioned in Materials and Methods for PET imaging)? Since the heart levels for anti-hTfR.B1 is lower than the control IgG, correcting the brain levels of the two antibodies against the heart levels would always give a higher value for anti-hTfR.B1. That is, the results shown in Fig 3D could be an artifact of the heart correction (Fig 3E) and the actual brain levels of anti-hTfR.B1 might not be that different than control IgG. To avoid this confusion, can the authors provide the uncorrected values for brain levels?

In PET measurements of the brain, it is common practice to correct for the blood volume present in the brain. About 5% of the total brain volume represents blood. Since radioactivity concentration in the blood is considerable, the correction is performed to obtain an accurate estimation of the radioactivity concentration in the brain tissue. Figure 3F was added to include the brain radioactivity concentrations which were not corrected for blood contribution. Figure 3G was updated and now includes PET images of the brain and the heart. PET images were not corrected for blood volume. It can be observed in Figure 3F and 3G that the radioactivity concentration in brain is higher for anti-hTfR-B1 than control IgG, even when no blood volume correction is applied for the brain.

6. In Fig 6, the authors show in vitro and in vivo correlation of various anti-mTfR antibodies. For the brain concentration (in vivo), do the authors have evidence that the concentration is mostly in the brain parenchyma and not brain vessels? In Karaoglu 2018 paper (Reference #9), immunohistology images of anti-mTfR and DVD-Igs containing anti-mTfR domain show that the concentration is not only from vasculature but also from molecules that penetrated brain parenchyma.

7. The hCMEC/D3 model failed to discriminate transcytosis of anti-hTfR vs control antibodies. Can the authors confirm if their cells still express the hTfR? Yes, hCMEC/D3 expresses human TfR. FACS data added (Figure 1C).

Reviewer #2: This manuscript submitted by Kangwen Deng et al. evaluated transcytosis of anti-human transferrin receptor (TfR) antibodies using several in vitro models derived from human, cynomolgus, and mouse species. The authors found that models with low barrier properties (low TEER), including hCMEC/D3 and bEnd3, were not suitable for in vitro transcytosis assays. Meanwhile, the authors indicated that Caco-2, a monkey brain endothelial cell-based model, and mIEC, which have high barrier properties, were appropriate as in vitro transcytosis models.
Globally, the study appears to be well performed, and the results are interesting. Thus, some points need to be addressed by the authors to improve the quality of the article.

1. Statistics
The authors should discuss this based on the statistical results, even if they are obvious.
Please perform statistical analysis and describe the statistical methods and results.
The Stats were added to all data
2. Figure 3F.
Please show a PET image of the heart for comparison with that of the brain. Figure 3G was updated and now includes PET images of the brain and the heart.


3. Comparative data on TfR protein expression levels in each model might be useful for understanding the differences in RMT transcytosis between models. Did the author examine expression levels in each model? Figure 7 - TfR protein expression in primary brain endothelial cells and cells that were used in in vitro transcytosis model, was added.

4. Discussion section (p17 lane 12)
For anti-hTfR.B1, its maximal transcytosis rate in Caco-2 and in MBT is 0.4 nmol/cm2/h and 13 nmol/cm2/h respectively…..
Please also show the data as the fold-over IgG controls. The MBT model might have higher permeability through the paracellular route than the Caco-2 model because of the difference in tight junction functions (TEER value).

Background paracellular route transport has been subtracted from this calculation. The fold-over IgG controls vary at different input conditions. For instance, referring to the data in Figure 3B, the fold over IgG at 1, 3, 10, 30 ug/ml is 93x, 24x, 10x and 4x respectively. The rate of anti-TfR.B1 transport remains the same when anti-TfR.B1 input is greater than 3 ug/ml. A sentence describing how the data was obtained was added to the text.

5. Please check the following points.
1) Figure 5A, Y-axis: ohm/cm2 => ohm x cm2 Figure was corrected.
2) Discussion, p17 Lane 16: Optimal in vitro transcytosis (Figure 4C) …=> I could not find Figure 4C That should be Figure 2D. The text was corrected.
3) Figure 3 legend, p23: …NHP using PE€(E) => PET The text was corrected.
